# Back to Nature: Combating *Candida albicans* Biofilm, Phospholipase and Hemolysin Using Plant Essential Oils

**DOI:** 10.3390/antibiotics10010081

**Published:** 2021-01-15

**Authors:** Ahmed M. El-Baz, Rasha A. Mosbah, Reham M. Goda, Basem Mansour, Taranum Sultana, Tanya E. S. Dahms, Amira M. El-Ganiny

**Affiliations:** 1 Microbiology and Biotechnology Department, Faculty of Pharmacy, Delta University for Science and Technology, International Coastal Road, Gamasa City, Mansoura, 11152 Dakhaliya, Egypt; elbaz_pharmacy@yahoo.com (A.M.E.-B.); Dr_reham_magdy@yahoo.com (R.M.G.); 2 Infection Control Unit, Zagazig University Hospitals, 44519 Zagazig, Egypt; rashamosbah@hotmail.com; 3 Pharmaceutical Chemistry Department, Faculty of Pharmacy, Delta University for Science and Technology, International Coastal Road, Gamasa City, Mansoura, 11152 Dakhaliya, Egypt; basem2412@yahoo.com; 4 Department of Chemistry and Biochemistry, University of Regina, Regina, SK S4S 1P4, Canada; taranum.sultana0@gmail.com (T.S.); tanya.dahms@uregina.ca (T.E.S.D.); 5 Microbiology and Immunology Department, Faculty of Pharmacy, Zagazig University, 44519 Zagazig, Egypt

**Keywords:** *Candida albicans*, essential oils, jasmine oil, biofilm, SEM, AFM, phospholipase, hemolysin, molecular docking

## Abstract

*Candida albicans* is the causative agent of fatal systemic candidiasis. Due to limitations of antifungals, new drugs are needed. The anti-virulence effect of plant essential oils (EOs) was evaluated against clinical *C. albicans* isolates including cinnamon, clove, jasmine and rosemary oils. Biofilm, phospholipase and hemolysin were assessed phenotypically. EOs were evaluated for their anti-virulence activity using phenotypic methods as well as scanning electron microscopy (SEM) and atomic force microscopy (AFM). Among the *C. albicans* isolates, biofilm, phospholipase and hemolysins were detected in 40.4, 86.5 and 78.8% of isolates, respectively. Jasmine oil showed the highest anti-biofilm activity followed by cinnamon, clove and rosemary oils. SEM and AFM analysis showed reduced adherence and roughness in the presence of EOs. For phospholipase, rosemary oil was the most inhibitory, followed by jasmine, cinnamon and clove oils, and for hemolysins, cinnamon had the highest inhibition followed by jasmine, rosemary and clove oils. A molecular docking study revealed major EO constituents as promising inhibitors of the Als3 adhesive protein, with the highest binding for eugenol, followed by 1,8-cineole, 2-phenylthiolane and cinnamaldehyde. In conclusion, EOs have a promising inhibitory impact on Candida biofilm, phospholipase and hemolysin production, hence EOs could be used as potential antifungals that impact virulence factors.

## 1. Introduction

The incidence of fungal infections, including candidiasis, has increased worldwide over the past few decades [1]. *Candida albicans* remains the most common causative agent for candidiasis [2], leading to a range of life-threatening invasive or non-life-threatening superficial conditions [3]. During invasive infections, *Candida* have the ability to enter the blood and infect every organ in the host [4]. Candida represents the fourth leading cause of nosocomial infections and the third most common cause of catheter-related bloodstream infections, with a mortality rate exceeding 50% [5,6].

Treatments of fungal infections have improved in the past decades with the introduction of new antifungals, but with modest success in reducing the high mortality rates associated with invasive infections [5]. Furthermore, the treatment of fungal infections is complicated by the emergence of resistance [7], which necessitates the development of novel therapeutic strategies or finding new antifungals. Targeting virulence mechanisms is an attractive option for discovering new antifungals by targeting microbial virulence without affecting cell viability, which may be less prone to the development of drug resistance [8]. Many virulence factors, including biofilm formation and production of hydrolytic enzymes, contribute to the pathogenesis of *Candida* [9,10]. Screening new drugs for their impact on virulence is an important tool for exploring novel antifungal targets leading to improved therapeutic regimens [10].

The first event in candida infection is adherence to the host or medical-device surfaces leading to biofilm formation; the cell wall proteins Als1, Als3, and Hwp1 are essential for adherence during biofilm development [9]. The increase in Candida infections in recent decades is almost paralleled to the widespread use of medical implant devices [11]. Biofilms not only cause colonization of implanted devices, adversely affecting their function, but also contribute to high antifungal resistance and escape from host defenses, which results in persistent infections [12].

Extracellular hydrolytic enzymes including phospholipases and hemolysins play critical roles in the pathogenesis of *C. albicans* by enhancing survival in the host [13]. Phospholipases facilitate invasion of the host epithelia by hydrolyzing phospholipids [14], while hemolysins degrade red blood cells and extract elemental iron, and both key virulence factors promote persistence in the host [15].

Various naturally derived molecules including essential oils (EOs) have shown antimicrobial activities against bacteria, fungi and even viruses including COVID-19 [16,17]. Many EOs have the potential to be used as antifungal agents [18,19]. For example, clove and cinnamon oils exhibited strong activity against fluconazole-resistant *C. albicans* strains [20], rosemary oil showed promising antifungal effects against *C. albicans* and *Aspergillus niger* [21], while jasmine oil has antifungal activity against dermatophytes [22]. The antimicrobial activity of plant oils is mainly attributable to a number of phenolic, alcoholic and terpenoid compounds [18]. Recent studies have evaluated the possibility of using EOs, not directly as biocidal agents, but as agents targeting *Candida* virulence [23,24,25], however the anti-virulence potential of EOs has not been extensively explored and requires further investigation.

The aim of this work is to study some virulence factors of *C. albicans* isolates, including biofilm formation and hydrolytic enzymes secretion, and to evaluate sub-inhibitory concentrations of clove oil, cinnamon oil, jasmine oil and rosemary oil as possible anti-virulence agents. The impact of EOs on *C. albicans* was assessed using traditional microbiological techniques, as well as scanning electron microscopy (SEM) and atomic force microscopy (AFM). The results presented here clearly demonstrate the anti-virulence potential of tested EOs, showing promising inhibition of biofilm, hemolysin and phospholipase production by clinical *C. albicans* isolates.

## 2. Results

### 2.1. Identification of C. albicans Isolates

**Figure 1:**
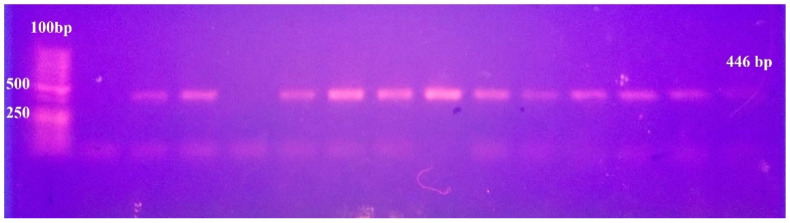
Confirmatory PCR for identification of *C. albicans* isolates. Lane 1: 100 bp ladder, lanes 2, 4 showed negative results, while the rest of lanes had a PCR products of *C. albicans* isolates with a band of 446 bp.

From a total of 74 clinical *Candida* isolates, 52 (70.3%) were identified as *C. albicans* with 30 from the respiratory tract infections (RTI), 16 from the urinary tract infections (UTI) and six from blood infection. Forty-eight *C. albicans* isolates developed a germ tube after incubation in serum for 2 h at 37 °C, and 51 developed green colonies on Chromogenic agar. Identification was confirmed by PCR, for which a single band of 446 bp appeared for 52 isolates (Figure 1).

### 2.2. MIC of Essential Oils Against C. albicans

The minimum inhibitory concentrations (MICs) of four essential oils were determined by the broth micro-dilution method. Cinnamon oil had the highest antifungal activity, with MICs ranging from 64–500 µg/mL and a MIC_50_ of 250 µg/mL. Clove, jasmine and rosemary oils had MIC_50_ of 500 µg/mL, with MICs ranging from 16–2000 µg/mL for jasmine and rosemary oils and 64–2000 µg/mL for clove oil (Table 1). The MICs of the tested oils were also determined for the standard strain *C. albicans* ATCC10231 and are shown in Table 1.

### 2.3. Screening for Virulence Factors of C. albicans Isolates

The ability of *C. albicans* to form biofilm was quantified using the CV assay for which 21 isolates were biofilm-positive, with 11 strong biofilm producers, one moderate and nine weak (Table 2), and eight out of the 11 (72%) strong biofilm isolates were from UTI, two from RTI and one from blood.

Phospholipase and hemolysin screens showed that the *C. albicans* isolates varied in the number of enzymes produced. For phospholipase, 45 isolates (86.5%) were positive; among them, 14 isolates (26.9%) were strong producers, 14 (26.9%) were intermediate and 17 (32.7%) were weak producers (Table 2). Forty-one isolates (78.8%) were hemolysin producers, with only six isolates showing strong activity, 26 intermediate and nine had weak activity (Table 2). *C. albicans* ATCC10231 was weak in biofilm formation, a moderate hemolysin producer (Hz = 0.8), while phospholipase activity was not detected in this strain.

### 2.4. Inhibition of Virulence Factors Using Sub-MIC of EOs

For each assay, six strains having the strongest activity were selected to test the anti-virulence effect of EOs at ¼ MIC. The tested concentration had no significant effect on *C. albicans* growth and viability, based on measuring the turbidity (OD_600_) after overnight growth with and without ¼ MIC of EOs (Supplementary Figure S1) and by viable counts of EOs-treated and untreated cells (Supplementary Figure S2).

#### 2.4.1. Biofilm Inhibition

**Figure 2:**
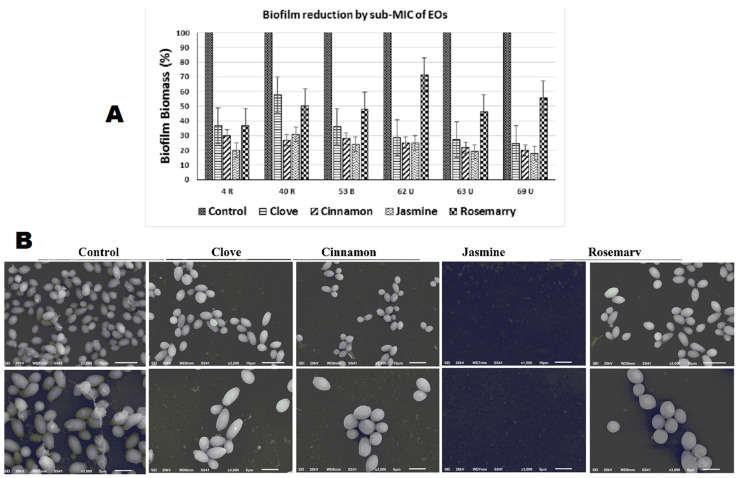
Assessment of biofilm and adhesion inhibition in clinical *C. albicans* isolates in absence (control) and presence of sub-MIC of cinnamon, clove, jasmine and rosemary oils, using (**A**) crystal violet assay of biofilm biomass showing significant reduction in absorbance upon exposure to essential oils (EOs). (**B**) SEM images of control *C. albicans* and those exposed to EOs, scale bars are 10 µm in upper panel and 5 µm in lower panel.

Jasmine oil was found to have the strongest anti-biofilm activity, followed by cinnamon, clove and rosemary oil, respectively (Figure 2A). Among the six strong biofilm forming isolates, four isolates no longer produced biofilm and two became weak producers in the presence of jasmine oil at ¼ MIC. With cinnamon oil, three isolates became non-producers and three became weak biofilm formers. For clove oil, one isolate became a non-producer, four became weak and one became a moderate biofilm producer, and for rosemary oil, three isolates became weak and three became moderate. All isolates had significantly (*p* < 0.05) lower absorbance values than control (Table 3).

SEM images, used to elucidate the effect of EOs on *Candida* attachment for biofilm formation, showed results that were consistent with that of the CV assay. Adhered cells were almost completely absent with jasmine oil exposure, there was a reduction in the number of attached cells with clove and cinnamon oil treatment, while rosemary oil had the greatest number of adhered cells (Figure 2B).

**Figure 3:**
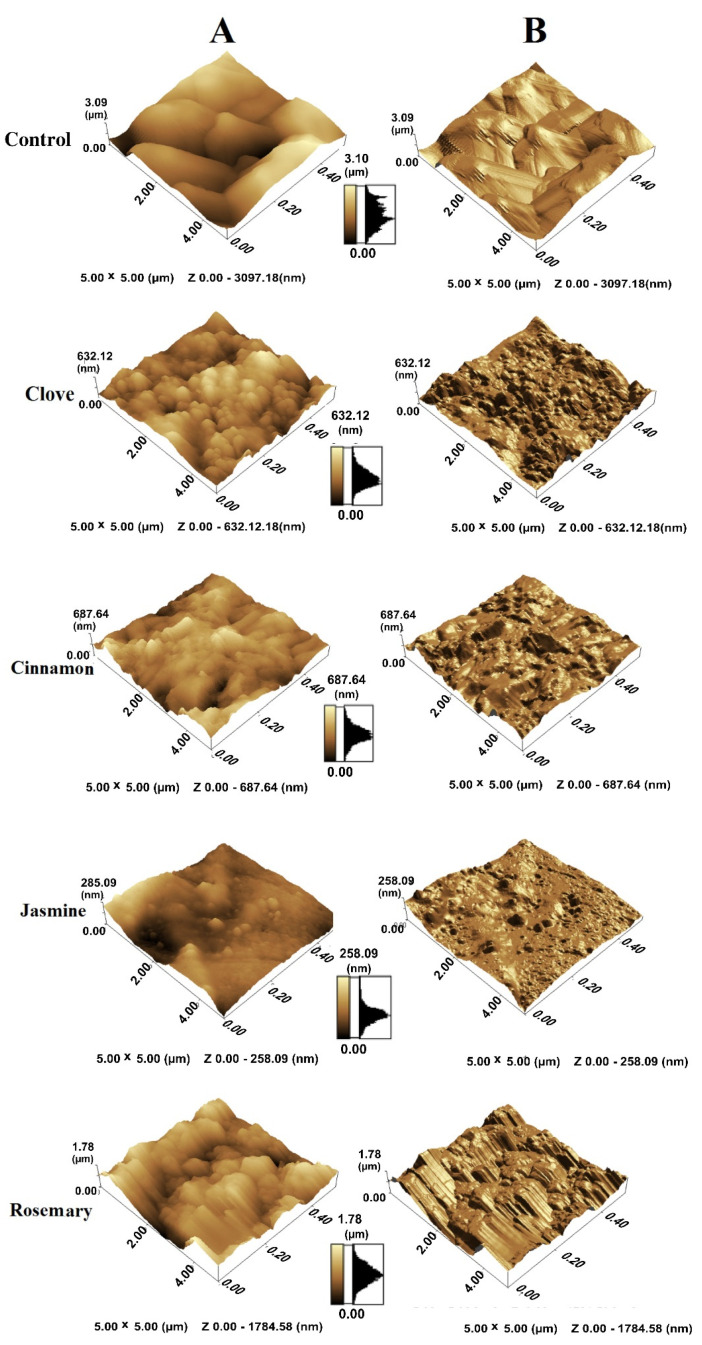
Atomic force microscopy 3D-topography images showing changes in cell height (**A**) and surface roughness (**B**) of *Candida albicans* control cells and EO-treated cells. Scanning area size is 5 × 5 µm.

AFM analysis showed variation in cell surface roughness and height in the presence of EOs (Figure 3). A significant reduction in the cell height (Z value) was observed, from 2332 ± 450 nm for control to 381 ± 80, 438 ± 80, 117 ± 25 and 1287 ± 190 nm with exposure to ¼ MIC of clove, cinnamon, jasmine and rosemary oils, respectively (Figure 3A). The roughness value was 2.46 ± 0.98 for control cells and was reduced in the presence of ¼ MIC of EOs to be 1.74 ± 0.62, 1.48 ± 0.62, 1.72 ± 0.7 and 2 ± 0.63 for clove, cinnamon, jasmine and rosemary oils, respectively (Figure 3B).

#### 2.4.2. Phospholipase Inhibition

**Figure 4:**
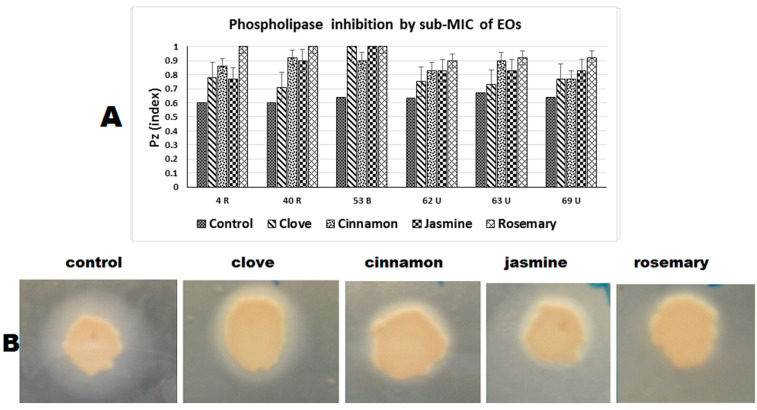
Phospholipase inhibition in *C. albicans* clinical isolates. (**A**) Graph showing changes in Pz value upon exposure to different EOs (the smaller the Pz value, the stronger the phospholipase activity and vice versa). (**B**) Representative images of phospholipase inhibition on egg-yolk agar containing sub-MIC of EOs.

Phospholipase inhibition using sub-MIC of EOs against six of the strong phospholipase-producing isolates are shown in Table 3. Rosemary had the highest activity, with three strains showing complete phospholipase inhibition, and three becoming weak producers. For cinnamon oil, three isolates became weak and three became moderate producers, while for jasmine oil, one isolate became a non-producer, two became weak and three became moderate. Finally, clove showed the lowest inhibitory effect with only one isolate becoming weak and five isolates becoming moderate (Figure 4).

#### 2.4.3. Hemolysin Inhibition

**Figure 5:**
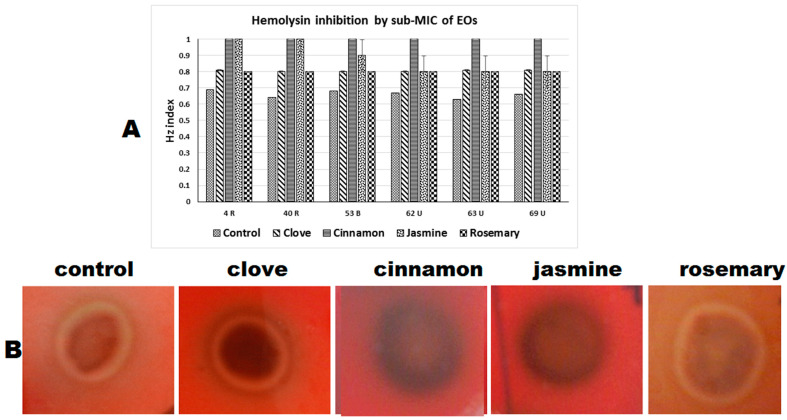
Hemolysin inhibition in *C. albicans* clinical isolates. (**A**) Graph showing changes in Hz value upon exposure to sub-MIC of different EOs (the smaller the Hz value the stronger the hemolysin activity and vice versa). (**B**) Representative images of hemolysin inhibition on Sabouraud dextrose-blood agar containing sub-MIC of EOs.

Cinnamon oil exposure had the highest inhibitory effect on hemolysin, with all tested strains becoming negative. Jasmine oil showed good inhibition, with two strains becoming negative, one weak and three showing moderate hemolysin activity. For clove and rosemary oil exposure, all the tested strains showed moderate hemolysin activity in the presence of both oils at ¼ MIC (Table 3, Figure 5).

#### 2.4.4. Molecular Docking of EOs Major Constituents with Als3 Protein

**Figure 6:**
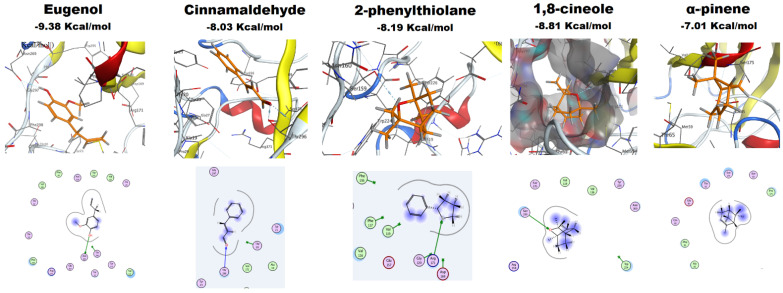
The putative binding mode (2D and 3D) with the mol-dock scores of Eugenol (clove oil); Cinnamaldehyde 2-Phenylthiolane and rosemary major constituents; 1,8-cineol; α-pinene into the pocket of the *C. albicans* Als3 surface protein.

The docking results of the major constituents of clove oil (eugenol), cinnamon oil (cinnamaldehyde), jasmine oil (2-phenylthiolane) and rosemary oil (1,8-cineole and α-pinene) into the Als3 surface protein are shown in Table 4 and Figure 6. For Eugenol, its OH group acts as a hydrogen donor, having an H-bond with Asn269, steering the ligand to achieve a stable complex with the receptor. Docking of cinnamaldehyde into the protein pocket revealed that the ligand’s formyl oxygen atom is capable of a bifurcated H-bond with the conserved amino acid Thr296 in the core of the hot spot of Als3. Furthermore, docking of 2-phenylthiolane revealed an interaction between the ligand and the active site, with a high docking score of −8.19 Kcal/mol. The sulfur atom in the thiolane ring serves as a hydrogen acceptor, forming an H-bond with the conserved amino acid Arg171. Furthermore, the hydrophobic and hydrophilic interactions improved the overall binding and recognition.

For 1,8-cineole, the electronegative hetero oxygen atom has a conspicuous role in the formation of a H-bond with the conserved hydrogen donor amino acid Val172 (peptide chain amide) at the core of the active site of the receptor. The electrostatic interactions improved the stability of ligand/receptor complex. Docking results for α-pinene showed no H-bond, which can be ascribed to the absence of heteroatoms in this essential oil component, but the hydrophilic and hydrophobic interactions found between the ligand and the active site of Als3 protein drive the stability of the ligand/receptor complex, with a significant binding free energy score.

## 3. Discussion

The treatment of fungal infections has become problematic based on the limited number of antifungals and the emergence of resistance to available drugs. There is a need to identify new antifungals or new therapeutic strategies [8]. Recently, there has been increased scientific interest in the biological properties of EOs that represent a safe alternative, with few side effects. EOs have been historically used for the treatment of infectious diseases in traditional medicine worldwide [26]. Most previous studies have concentrated on the antimicrobial activity of EOs [18,21,27,28,29], but in the current study, we examined the anti-virulence effect of four EOs on clinical *C. albicans* isolates.

*C. albicans* remains the fungal species most commonly associated with biofilm formation. Most clinical manifestations of candidiasis are linked to biofilm formation on implanted medical devices [11]. In this study, about 40.3% of *C. albicans* isolates were capable of forming biofilms. Interestingly, 13 out of the 16 isolates from UTI (81%) were biofilm-forming, eight of which were strong biofilm producers, consistent with reports that the widespread use of indwelling urinary catheters is one of the main correlates to urinary candidiasis [30]. About 23% of the RT isolates in our study were biofilm forming, consistent with biofilm in intubated RT patients [31]. Only one out of six blood isolates (16%) was a biofilm producer.

The biofilm-forming *C. albicans* isolates are much more resistant to antifungals [12,32]. Hence, new agents that can inhibit biofilm formation are urgently needed and would enhance therapeutic efficacy. Our results showed that jasmine oil has strong anti-biofilm and anti-adhesion activity, followed by cinnamon, clove and then rosemary oil. All of these oils were able to prevent or reduce biofilm formation when used at sub-inhibitory concentrations, consistent with evidence that cinnamon oil inhibits the growth of *C. parapsilosis* and *C. albicans* biofilms with a MIC of 250 and 125 µg/mL, respectively [33]. Further, Agarwal et al. reported that plant EOs, including clove oil, have strong antifungal and anti-biofilm activities against *C. albicans* [34]. The strong anti-biofilm activity of EOs make them excellent candidates for coating biomaterials, a strategy that significantly reduces biofilm formation [35]. For example, coating catheter materials with camphor-containing films significantly reduced biofilm formation by *C. albicans* strains [36].

Hemolysin and phospholipase are hydrolytic enzymes important for the virulence of *C. albicans*. The majority of isolates (86.5%) showed phospholipase activity consistent with previous studies demonstrating *C. albicans* to be a good phospholipase-producing species [13,37,38]. On the other hand, hemolytic activity was observed in only 78.8% of our *C. albicans* isolates, lower than the previously reported at 94.8% [38]. Since the absence of glucose in the culture medium reduces hemolytic activity [39], the higher percentage reported by Sachin and his colleagues can be attributed to their higher glucose (3%) containing media [38].

Our blood isolates showed a high level of phospholipase production (100%), followed by respiratory isolates (86.7%) and then urine isolates (81%). For hemolysin production, isolates from blood and respiratory aspirates had the same activity (83%), while urine isolates showed lower activity (69%). It has been reported that the quantity of phospholipase produced by *Candida* correlates with the site of infection [40]. Price and coworkers reported that blood isolates generally produce much higher levels of phospholipase than do isolates from wounds or urine [41], while other studies reported that phospholipase positivity was higher in respiratory isolates than blood and urine [42,43]. The variable outcomes may relate to the different number of tested strains from each site of infection in the different studies.

Regarding phospholipase inhibition, rosemary oil had the highest activity followed by cinnamon, jasmine and clove oils, respectively. While for hemolysin inhibition, cinnamon oil had the greatest effect, followed by jasmine, clove and rosemary oils. The work of Budzyńska and coworkers showed that lemon balm, citronella, geranium and clove oils at sub-MIC reduced enzymatic activity, including phospholipase and hemolysins of reference *C. albicans* strains [23].

The differential activity of tested EOs against biofilm, phospholipase and hemolysin may be related to the chemical composition of these EOs. Several studies [44,45,46] show that the major compound of cinnamon bark oil is cinnamaldehyde (52–80%), the main constituent in clove oil is eugenol with concentration ranges from 68 to 88% [21,47,48], for rosemary oil, 1,8-cineole (26.5–27.2%) and α-pinene (19.2–20%) are predominant [21,49], while jasmine oil has 57.3% of 2-phenylthiolane [50]. The EO antifungal activity can often be attributed to the major constituent [44], or from synergistic effects between two or more EO components [29].

Cinnamaldehyde and eugenol are phenylpropanoids, and their addition during the early phase of biofilm formation, immediately following adhesion, prevents biofilm development. Cinnamaldehyde was more potent than the quorum-sensing molecule farnesol in inhibiting biofilm development, while eugenol has similar potency as farnesol [51], which is consistent with our results showing that cinnamon oil is more powerful than clove oil in inhibiting biofilm. Further, cinnamaldehyde has a reported inhibitory effect on a plasma membrane ATPase, which is involved in hydrolytic enzyme secretion [52]. Of the monoterpenes 1,8-cineole and α- pinene from rosemary oil, α-pinene proved to be a very active compound, while 1,8-cineole has lower antifungal activity [53]. Additionally, the monoterpenoid β-citronellol decreases the hydrolytic enzymes secretion in *C. albicans* [54], consistent with the strong inhibition of phospholipase by rosemary oil in our study.

Very little information regarding the antifungal activity of jasmine oil exists in the literature. A previous study showed that jasmine caused 100% inhibition of spore germination of oto-mycotic pathogens, including *C. albicans* [55]. Further, jasmine EO showed promising inhibitory effects against *Malassezia species* isolated from skin infections [22], as well as oral strains of *Candida species* [56], and a recent study reported its fungicidal activity against mold fungi [57]. However, none of these studies evaluate the anti-virulence potential of jasmine oil. To our knowledge, the current study is the first to assess the anti-virulence activity of jasmine oil on clinical *C. albicans* isolates, with only one previous study showing its anti-biofilm activity against bacterial isolates from eye infections [58]. Here, we demonstrate that jasmine oil has outstanding anti-biofilm activity and a moderate inhibitory effect on phospholipase and hemolysin production.

Previous studies highlighted the promising effect of EOs that serve as the basis for their use in the treatment of candidiasis in different infection sites including RTIs and superficial infections. Spray applications of some EOs have significantly improved the symptoms of upper respiratory infections [29,59]. A recent study revealed that cinnamon and clove oils showed significantly higher efficacy than miconazole against vulvo-vaginal *C. albicans* isolates [60]. Additionally, clove oil had a promising in vitro activity against the dermatophyte *Microsporum spp* and showed low toxicity to mammalian cell lines [48]. However, further clinical trials are required to establish EOs-safety, efficacy and the most suitable drug delivery system.

SEM and AFM analyses help rationalize the structural bases of interactions between drugs and pathogens on the nanoscale and can facilitate screening new therapeutic agents [61,62,63]. With sub-MIC EO exposure, SEM analysis showed altered cell adhesion that was accompanied by a reduction in cell height and roughness as determined by AFM imaging, explaining the reduced biofilm formation in terms of reduced adhesion, the first step in biofilm formation. A previous study analyzed the effect of honey on candida biofilm using SEM and AFM and reported changes in the cellular morphology of *C. albicans* and a reduction in biofilm thickness [64]. Another study used AFM to show that EOs’ nanoparticles reduced *Candida species* biofilm height [65]. A recent study by our group used AFM to analyze the impact of cinnamon bark oil on *Candida albicans* at sub-MIC, revealing changes in cell surface roughness, cell wall adhesion and elasticity [46]. Additionally, it has been reported that *C. albicans* adhesion tends to increase with increased surface roughness [66], consistent with our results showing reduced roughness accompanied by a reduction in biofilm formation.

The adhesive phenotype of *C. albicans* contributes to its ability to colonize the host and also plays a vital role in biofilm formation. The Als (agglutinin-like sequence) adhesive proteins are one of the most widely studied *C. albicans* virulence attributes, with deletion of Als3 producing the greatest reduction in adhesive function [67]. The results of molecular docking to Als3 protein suggest that the tested compounds have an impact on the Als3 protein and can be considered promising inhibitors. Similarly, a previous study reported that thiazolidinedione derivatives found to be biofilm inhibitors act by obstructing the functions of the *C. albicans* Als surface proteins [68]. The highest binding score was reported for eugenol, followed by 1,8-cineole, 2-phenylthiolane and cinnamaldehyde, and with α-pinene showing the lowest score. Understandably, the order of mol-dock scores for the five compounds did not follow the same order as the biological activities of EOs as biofilm inhibitors, which may relate to several mechanisms playing roles in the adhesion process. Future docking studies using other Als family proteins and all possible targets for these five compounds will be pursued.

## 4. Materials and Methods

### 4.1. Isolation and Identification of C. albicans Isolates

Clinical isolates of *C. albicans* were isolated from Mansoura and Zagazig University hospitals in Egypt, the isolates were obtained from the hospital clinical laboratories and not directly taken from patients. The isolates recovered from respiratory aspirates, blood and urine samples were phenotypically identified as *C. albicans* by germ tube test in human serum and by the growth characteristics on Chromogenic agar (Pronadisa, Madrid, Spain). Strains were further identified by the polymerase chain reaction (PCR) using the species-specific primers (Sigma Aldrich, St Louis, MO, USA): Calb-F 5′-AGCTGCCGCCAGAGGTCTAA-3′ and Calb-R 5′-TTCTTTTCCTCCGCTTATTG-3′ [69]. Strains were maintained in 50% glycerol stocks frozen at −70 °C and streaked on Sabouraud dextrose agar (SDA) plates (Oxoid, Hampshire, England) to obtain an overnight culture prior to each experiment.

### 4.2. Minimum Inhibitory Concentrations (MICs) Determination for EOs

The essential oils tested were *Syzygium aromaticum*; clove (El-Nasr Pharmaceutical Chemicals Company (ADWIC), Abou-Zabel, Egypt), *Jasminum grandiflorum*; jasmine (Morgan Chemicals Company, Cairo, Egypt), *Cinnamomum verum*; cinnamon and *Rosmarinus officinalis*; rosemary (locally extracted in the Pharmacocgnosy Department, Faculty of pharmacy, Zagazig university using Clevenger type hydro-distillation extractor).

The MICs of essential oils were determined using the broth micro-dilution method [70], with some modifications. Briefly, stock solutions of essential oils (8 mg/mL) were prepared in yeast peptone dextrose (YPD) broth with the addition of 0.5% *v/v* Tween 80 to help homogenous dissolution of EOs in the media. Twofold serial dilutions of these oils were prepared in YPD in 96-well microtiter plates, so the concentration range in the wells of microtiter plate was 4–4000 µg/mL. *C. albicans* isolates were cultured on YPD agar medium at 37 °C for 48 h. A suspension of each isolate was prepared, and the turbidity was adjusted to 0.5 McFarland turbidity standard (equivalent to 1–5 × 10^6^ CFU/mL) and diluted 1/100 in YPD broth. Diluted suspension (100 µL) was added to each well containing 100 µL of various oil concentrations to give a final inoculum concentration 1–5 × 10^4^ CFU/mL. The MIC was recorded after 48 h of incubation at 37 °C and defined as the lowest concentration that showed no growth estimated by visual inspection. The experiment was performed in triplicate.

### 4.3. Screening and Inhibition of C. albicans Virulence Factors

The anti-virulence effect of EOs was tested at ¼ MIC. To confirm that this concentration had no significant effect on *C. albicans* viability, it was grown overnight in YPD broth with and without EOs at ¼ MIC. Following overnight incubation at 37 °C, the optical densities of EOs-treated cultures and untreated cultures were measured at 600 nm (OD_600_) by *spectrofluorometer* (Biotek, Winooski, VT, USA). Moreover, the viable counts after 24 h incubation at 37 °C were determined [46]. The inhibitory effect of ¼ MIC of EOs was tested on biofilm, phospholipase and hemolysin production.

#### 4.3.1. Biofilm Formation and Inhibition

The ability of *C. albicans* isolates to form biofilm was evaluated using the crystal violet assay (CV) in 96 well microtiter plates, with some modification [71]. The CV assay provides an overall assessment of biofilm biomass. Briefly, a fresh aqueous yeast suspension of 0.5 McFarland turbidity was prepared from an overnight culture and diluted 1/100 in YPD broth. Aliquots of 200 μL of standardized inoculum suspension were distributed in a 96-well microtiter plate (4 wells for each strain) and incubated under stationary conditions for 48 h at 37 °C, with wells containing media only included as negative controls. The content of each well was aspirated, the wells washed three times with PBS, fixed with 200 μL of 99% methanol for 15 min, decanted, air dried and stained with 200 μL of 2% CV for 15 min. Excess stain was gently removed by rinsing with water, the plates air-dried, and the bound dye solubilized with 200 μL of 33% glacial acetic acid. The optical density (OD) was measured at 590 nm using a microtiter plate reader Synergy HT (BioTek Instruments, Winooski, VT, USA). The reference strain *C. albicans* ATCC10231 served as a positive control. For each strain, the mean OD of four wells was calculated (ODt) and also cut-off OD (ODc) was defined as 3 standard deviations above the mean OD of the negative control. The level of biofilm production was determined as follows: non-biofilm producer (N) ODt ≤ ODc, weak biofilm producer (W) ODc < ODt ≤ 2 × ODc, moderate biofilm producer (M) 2 × ODc < ODt ≤ 4 × ODc and strong biofilm producer (S) ODt > 4 × ODc [72].

For testing the anti-virulence effect of EOs, ¼ MIC was used as a previous study reported that this concentration had no significant effect on *C. albicans* viability [24]. For the biofilm inhibition assay, the CV-microtiter plate method was used. The experiment was performed by the addition of 100 μL of sub-MIC EOs to wells containing 100 μL of standardized inoculum suspension in YPD. The plates were incubated under stationary conditions for 48 h at 37 °C, then processed, and the degree of biofilm formation in the presence of EOs was calculated as previously described [72].

#### 4.3.2. Phospholipase Activity and Inhibition

*C. albicans* isolates were screened for extracellular phospholipase activity using egg yolk agar [73]. Approximately 5 µL of *C. albicans* suspension, adjusted to 0.5 McFarland turbidity, was inoculated onto the egg yolk agar medium and left to dry at room temperature, and then incubated at 37 °C for 48 h. The diameter of the colony and the precipitation zone around the colony (indicator of phospholipase activity) were measured. The assay was conducted in triplicate for each isolate with the reference strain ATCC10231 as a positive control.

The phospholipase zone (Pz) index was defined as the ratio of the colony diameter to the total diameter of the colony, plus the precipitation zone. Phospholipase positive strains were classified according to Pz values, where Pz = 1 denotes a negative reaction, a value of 0.9–0.99 means weak activity, 0.89–0.70 moderate activity, while ≤0.69 indicates strong activity [40]. For testing the inhibitory effect of EOs, the experiment was repeated using egg yolk agar plates containing sub-inhibitory concentrations (¼ MIC) of EO and the Pz value was calculated again in the presence of EOs.

#### 4.3.3. Hemolysin Activity and Inhibition

Hemolytic activity of clinical *C. albicans* isolates was screened on SDA plates containing 5% sheep blood as described previously [39]. Approximately 5 μL of standard *Candida* suspension was inoculated onto the medium, culture plates were incubated at 37 °C for 48 h, and a zone of hemolysis around the colony indicated hemolysin production. The reference strain ATCC 10,231 served as the control. The hemolysin zone (Hz) index was calculated in a manner similar to Pz and used to classify the strains into weak, intermediate and strong producers [23]. For testing the inhibitory effect of EOs, the experiment was repeated using SDA/blood plates containing sub-inhibitory concentrations of essential oils (¼ MIC), and the Hz value was calculated again in the presence of EOs.

### 4.4. Scanning Electron Microscopy (SEM)

SEM was used to elucidate the effect of EOs on adhesion in the context of biofilm formation as described previously, with some modifications [34]. Briefly, biofilms were formed on Petri plates as previously described, and the supernatant was removed by aspiration. Small pieces of the plates were fixed with 2.5% glutaraldehyde in 0.15 M PBS for 1 h at room temperature. Samples were then treated with 1% osmium tetroxide for 1 h, washed three times with distilled water, treated with 1% uranyl acetate for 1 h, washed again with distilled water and dehydrated with a series of ethanol solutions that ranged from 30% to 100%. All samples were dried using a Tousimis Autosamdri 815 critical-point drier (Rockville, MD, USA) then gold coated on a SPI Module-Sputter Carbon/Gold Coater (West Chester, PA, USA) and imaged with a JEOL JSM 6510 SEM (Tokyo, Japan). The accelerating voltage was 20 kV, the beam current was 1.5 nA, for sample examination and 50 pA for image acquisition.

### 4.5. Atomic Force Microscopy (AFM)

Cells for AFM were established in 60 mm Petri plates in the presence and absence of sub-inhibitory concentrations of different EOs. After 48 h of incubation, the liquid medium was withdrawn, and the plates were washed twice with PBS, the samples fixed with 2.5% glutaraldehyde at 4 °C for 4 h, washed with distilled water and air dried [64]. All images were collected with a Wet-SPM-9600 scanning probe microscope (Shimadzu, Kyoto, Japan) in contact mode using Si_3_N_4_ cantilevers. The scanning area in the images was 5 × 5 μm with at least 20 different cells imaged for each biological replicate.

### 4.6. Molecular Docking Analysis

The crystal structure of *C. albicans* agglutinin-like sequence (Als3) protein (PDB: 4LEE, 2014) was retrieved from the protein data bank [74]. The docking module of Molecular Operating Environment (MOE version 2019.0102 Chemical Computing Group Inc., Montreal, QC, Canada) on a Pentium 1.6 GHz workstation with 512 MB memory and a windows operating system was used for docking studies [75]. The tested compounds were drawn into MarvinSketch using the Marvin suite [76], to generate images of the lowest energy conformer for each.

### 4.7. Statistical Analysis

The significance of differences between means for each virulence factor under variable conditions, data were analyzed by GraphPad Prism 7 (GraphPad Software Inc., San Diego, CA, USA) using one-way ANOVA (multiple comparisons). The level of significance was taken at a *p*-value ≤ 0.05.

## 5. Conclusions

The results presented here clearly demonstrate the anti-virulence potential of essential oils from selected plants, showing promising inhibition of biofilm, hemolysin and phospholipase production by clinical isolates of *C. albicans*. For the first time, we showed that jasmine oil has outstanding anti-biofilm activity and a moderate inhibitory effect on phospholipase and hemolysin production in *C. albicans* isolates. These results emphasize the possible applications of EOs in the clinical setting, either as coatings for biomaterials, as constituents of inhalers for upper RTI, or topically for treating superficial mycoses. Further in vivo studies will be pursued using animal models in preparation for the clinical use of EOs.

## Figures and Tables

**Table 1:** The MIC of essential oils against clinical strains of *C. albicans*.

Strains	ATCC 10231	Clinical Isolates (*n* = 52)	
Essential Oil	MIC (µg/mL)	MIC Range (µg/mL)	MIC_50_ (µg/mL)
Clove oil	125	64–2000	500
Cinnamon oil	250	64–500	250
Jasmine oil	500	16–2000	500
Rosemary oil	500	16–2000	500

**Table 2:** Number and percentage of *C. albicans* isolates from different clinical sources producing biofilm, phospholipase and hemolysin.

	Number (%) of *C. albicans* Producing		
	Biofilm	Phospholipase	Hemolysin
**According to degree of production**			
Strong (S)	11 (21.2%)	14 (26.9%)	6 (11.5%)
Moderate (M)	1(1.9%)	14 (26.9%)	26 (50%)
Weak (W)	9 (17.3%)	17 (32.7%)	9 (17.3%)
**According to source of strain**			
Respiratory (30)	7 (23.3%)	26 (86.7%)	25 (83%)
Urine (16)	13 (81%)	13 (81%)	11 (69%)
Blood (6)	1 (16.7%)	6 (100%)	5 (83%)
Total positive	21 (40.4%)	45 (86.5%)	41 (78.8%)

**Table 3:** Inhibition of *C. albicans* phospholipase, hemolysin and biofilm formation using sub-inhibitory concentration (¼ MIC) of different EOs.

Isolate No. (Source)	Control	Clove	Cinnamon	Jasmine	Rosemary
**Biofilm (absorbance (ODt) at 590 nm)**					
4 (R)	0.3 ^a^	0.11 ^b^	0.09 ^c^	0.06 ^c^	0.11 ^b^
40(R)	0.29 ^a^	0.15 ^b^	0.07 ^c^	0.08 ^c^	0.13 ^b^
53 (B)	0.29 ^a^	0.09 b	0.07 ^b,c^	0.06 ^c^	0.12 ^d^
62 (U)	0.3 ^a^	0.07 b	0.07 ^b^	0.07 ^b^	0.2 ^c^
63 (U)	0.37 ^a^	0.1 ^b^	0.08 ^b,c^	0.07 ^c^	0.17 ^d^
69 (U)	0.45 ^a^	0.11 ^b^	0.09 ^b^	0.08 ^b^	0.25 ^c^
**Phospholipase (Pz index)**					
3 (R)	0.6 ^a^	0.78 ^b^	0.86 ^c^	0.77 ^b^	1 ^d^
4 (R)	0.6 ^a^	0.71 ^b^	0.92 ^c^	0.9 ^c^	1 ^d^
20 (B)	0.64 ^a^	1 ^b^	0.9 ^c^	1 ^b^	1 ^b^
32 (B)	0.63 ^a^	0.75 ^b^	0.83 ^c^	0.83 ^c^	0.9 ^d^
56 (U)	0.67 ^a^	0.73 ^b^	0.9 ^c^	0.83 ^d^	0.92 ^c^
67 (U)	0.64 ^a^	0.77 ^b^	0.77 ^b^	0.83 ^c^	0.92 ^d^
**Hemolysin (Hz index)**					
4 (R)	0.69 ^a^	0.81 ^b^	1 ^c^	1 ^c^	0.8 ^b^
14 (R)	0.64 ^a^	0.8 ^b^	1 ^c^	1 ^c^	0.8 ^b^
46 (R)	0.68 ^a^	0.8 ^b^	1 ^c^	0.9 ^d^	0.8 ^b^
47 (R)	0.67 ^a^	0.8 ^b^	1 ^c^	0.8 ^b^	0.8 ^b^
65 (U)	0.63 ^a^	0.81 ^b^	1 ^c^	0.8 ^b^	0.8 ^b^
69 (U)	0.66 ^a^	0.81 ^b^	1 ^c^	0.8 ^b^	0.8 ^b^

R: respiratory; B: blood; U: urine; for biofilm, ODc = 0.07, non-producer (N) ODt ≤ ODc, weak (W) ODc < ODt ≤ 2 × ODc, moderate (M) 2 × ODc < ODt ≤ 4 × ODc and strong (S) ODt > 4 × ODc; for Pz and Hz index = 1(negative), 0.9–0.99 (weak), 0.89–0.70 (moderate), ≤ 0.69 (strong); ^a,b,c,d^ values followed by different letters are significantly different. Data analyzed by GraphPad Prism 7.0 using one-way ANOVA (multiple comparisons) at *p*-value < 0.05.

**Table 4:** Docking results for the major constituents of EOs into Als3 protein (PDB: 4LEE), indicating the putative binding free energy and potential hydrogen bonding residues.

EO	Major Constituents	Binding Free Energy (Kcal/mol)	H-Bond
Clove	Eugenol	−9.38	Asn 269
Cinnamon	Cinnamaldehyde	−8.03	Thr 296
Jasmine	2-phenylthiolane	−8.19	Arg 171
Rosemary	1,8-cineole	−8.81	Val 172
	α-pinene	−7.01	None

## Data Availability

The data presented in this study are available on request from the corresponding author.

## Supplementary Materials

The following are available online at https://www.mdpi.com/2079-6382/10/1/81/s1, Figure S1: The effect of essential oils on *C. albicans* growth. Figure S2: The effect of essential oils on *C. albicans* viability.
