# Supplementary material for: Back to Nature: Combating Candida albicans Biofilm, Phospholipase and Hemolysin Using Plant Essential Oils
Source: Antibiotics (Basel). 2021 Jan 15;10(1):81. doi: 10.3390/antibiotics10010081 (PMC7830859; doi:10.3390/antibiotics10010081)
Supplement: Supplementary file 1 [file antibiotics-10-00081-s001.pdf]

antibiotics

Article

Back to Nature: Combating *Candida albicans* Biofilm, Phospholipase and Hemolysin Using Plant Essential Oils

Ahmed M. El-Baz [1], Rasha A. Mosbah [2], Reham M. Goda [1], Basem Mansour [3], Taranum Sultana [4], Tanya E. S. Dahms [4] and Amira M. El-Ganiny [5,*]

[1] Microbiology and Biotechnology Department, Faculty of Pharmacy, Delta University for Science and Technology, International coastal road, Gamasa City, Mansoura, 11152 Dakhaliya, Egypt; elbaz_pharmacy@yahoo.com (A.M.E.-B.); Dr_reham_magdy@yahoo.com (R.M.G.)
[2] Infection control unit, Zagazig University hospitals, 44519 Zagazig, Egypt; rashamosbah@hotmail.com
[3] Pharmaceutical Chemistry Department, Faculty of Pharmacy, Delta University for Science and Technology, International Coastal Road, Gamasa City, Mansoura, 11152 Dakhaliya, Egypt; basem2412@yahoo.com
[4] Department of Chemistry and Biochemistry, University of Regina, Regina, SK S4S 1P4, Canada; taranum.sultana0@gmail.com (T.S.); tanya.dahms@uregina.ca (T.E.S.D.)
[5] Microbiology and Immunology Department, Faculty of Pharmacy, Zagazig University, 44519 Zagazig, Egypt
* Correspondence: amiraganiny@yahoo.com or AMElganiny@pharmacy.zu.edu.eg; Tel.: 002-010-22799736, Fax: 002-055-2303266

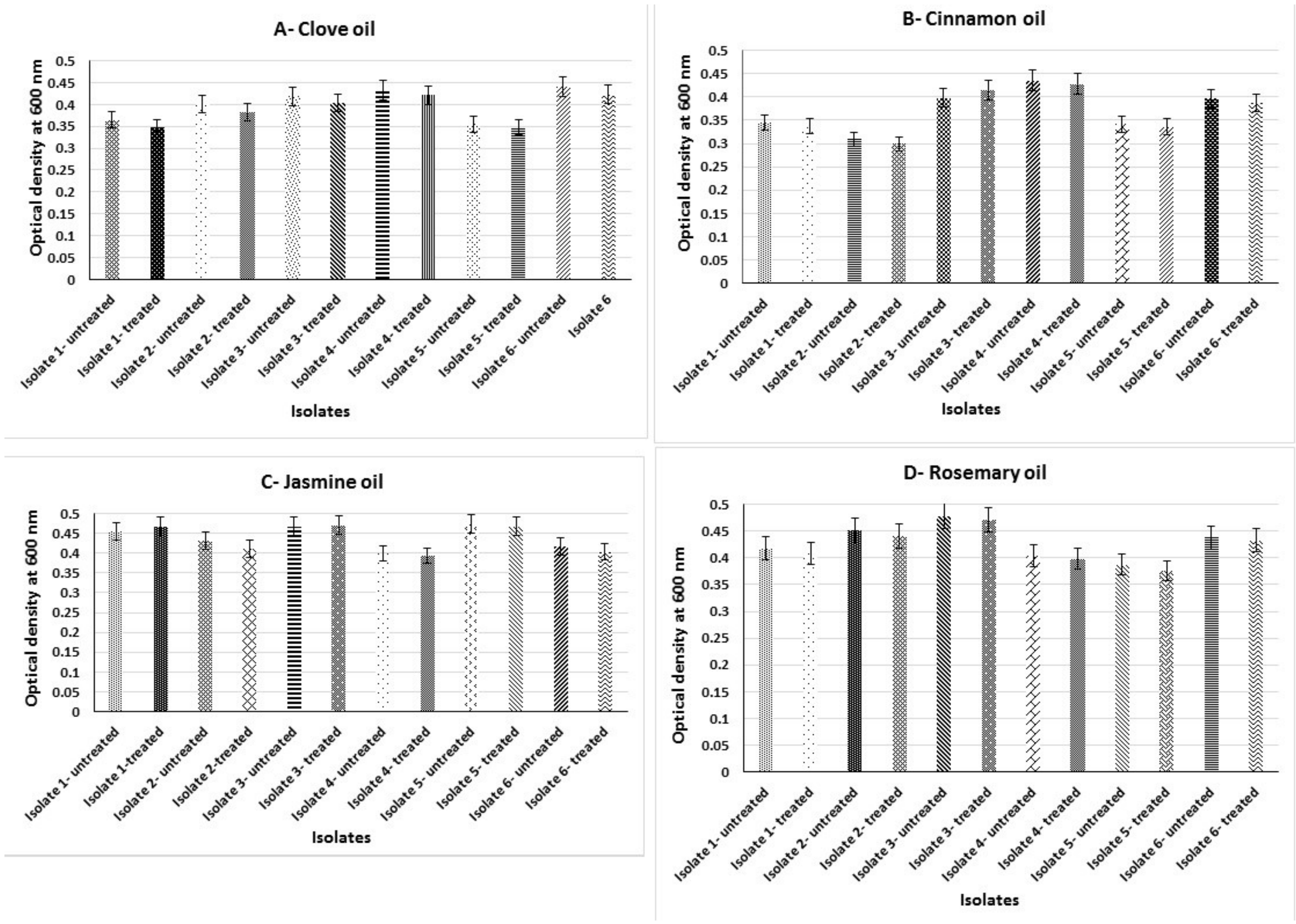

Figure S1. Effect of essential oils on *C. albicans* growth. The growth was assessed by measuring the OD600 of fungal suspensions after overnight incubation with ¼ MIC of **A**- Clove oil, **B**- Cinnamon oil, **C**- Jasmine oil, **D**- Rosemary oil. Comparison was done for EO-treated cultures and untreated ones, the test was done in duplicate. EOS had no statistically

significant inhibition of fungal growth. The data shown are the means± standard errors. Unpaired t test (two-tailed) was used for statistical analysis.

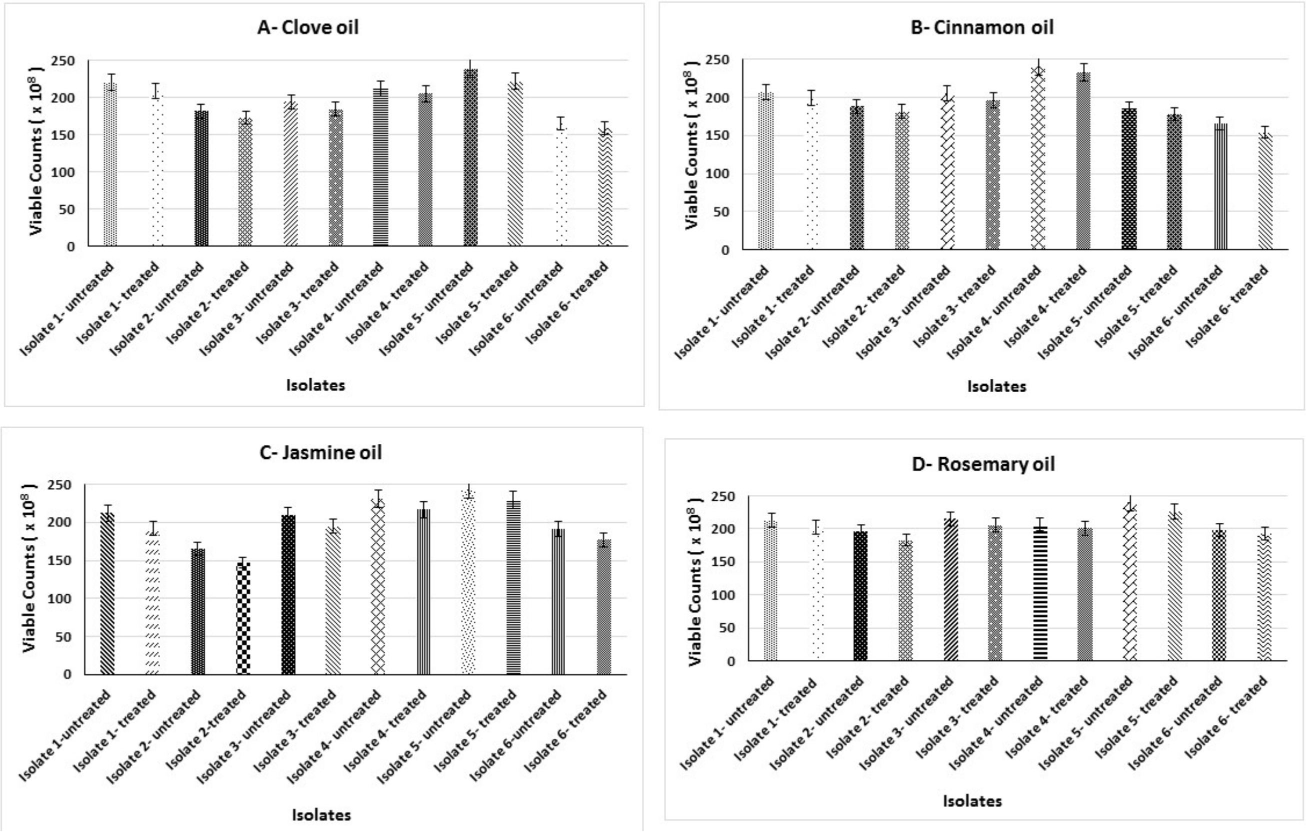

Figure S2. Effect of essential oils on *C. albicans* viability. The growth was assessed by measuring viable count after overnight incubation with ¼ MIC of **A**- Clove oil, **B**- Cinnamon oil, **C**- Jasmine oil, **D**- Rosemary oil. Comparison was done for EO-treated cultures and untreated ones, the test was done in duplicate. EOS had no statistically significant effect on viability of Candida cells. The data shown are the means± standard errors. Unpaired t test (two-tailed) was used for statistical analysis.
